# Patient educational level and management of bipolar disorder

**DOI:** 10.1192/bjo.2021.19

**Published:** 2021-03-08

**Authors:** Alina Karanti, Lana Bublik, Mathias Kardell, Kristina Annerbrink, Paul Lichtenstein, Bo Runeson, Erik Pålsson, Mikael Landén

**Affiliations:** Department of Psychiatry and Neurochemistry, Institute of Neuroscience and Physiology, Sahlgrenska Academy, University of Gothenburg, Sweden; Department of Psychiatry and Neurochemistry, Institute of Neuroscience and Physiology, Sahlgrenska Academy, University of Gothenburg, Sweden; Department of Psychiatry and Neurochemistry, Institute of Neuroscience and Physiology, Sahlgrenska Academy, University of Gothenburg, Sweden; Department of Psychiatry and Neurochemistry, Institute of Neuroscience and Physiology, Sahlgrenska Academy, University of Gothenburg, Sweden; Department of Medical Epidemiology and Biostatistics, Karolinska Institutet, Sweden; Department of Clinical Neuroscience, Centre for Psychiatry Research, Karolinska Institutet, Sweden; Department of Psychiatry and Neurochemistry, Institute of Neuroscience and Physiology, Sahlgrenska Academy, University of Gothenburg, Sweden; Department of Medical Epidemiology and Biostatistics, Karolinska Institutet, Sweden; and Department of Psychiatry and Neurochemistry, Institute of Neuroscience and Physiology, Sahlgrenska Academy, University of Gothenburg, Sweden

**Keywords:** Pharmacotherapy, psychotherapy, healthcare disparity, bipolar disorder, education

## Abstract

**Background:**

Socioeconomic factors can affect healthcare management.

**Aims:**

The aim was to investigate if patient educational attainment is associated with management of bipolar disorder.

**Method:**

We included patients with bipolar disorder type 1 (*n* = 4289), type 2 (*n* = 4020) and not otherwise specified (*n* = 1756), from the Swedish National Quality Register for Bipolar Disorder (BipoläR). The association between patients’ educational level and pharmacological and psychological interventions was analysed by binary logistic regression. We calculated odds ratios after adjusting for demographic and clinical variables.

**Results:**

Higher education was associated with increased likelihood of receiving psychotherapy (adjusted odds ratio 1.34, 95% CI 91.22–1.46) and psychoeducation (adjusted odds ratio 1.18, 95% CI 1.07–1.46), but with lower likelihood of receiving first-generation antipsychotics (adjusted odds ratio 0.76, 95% CI 0.62–0.94) and tricyclic antidepressants (adjusted odds ratio 0.76, 95% CI 0.59–0.97). Higher education was also associated with lower risk for compulsory in-patient care (adjusted odds ratio 0.79, 95% CI 0.67–0.93).

**Conclusions:**

Pharmacological and psychological treatment of bipolar disorder differ depending on patients’ educational attainment. The reasons for these disparities remain to be explained.

Bipolar disorder is a serious psychiatric condition associated with high societal costs.^1^ Besides pharmacotherapy, adjunct psychoeducation and psychotherapy can help prevent relapse and increase treatment adherence.

## Socioeconomic factors and risk for bipolar disorder

Socioeconomic factors, such as income, education and occupation, influence the risk for mental disorders.^2^ Interestingly, however, people with bipolar disorder more often belong to higher socioeconomic strata compared with controls or the general population.^3^ Likewise, a recent study suggested that higher socioeconomic status in parents was associated with increased risk of bipolar disorder in offspring.^4^ Educational attainment is a commonly used measure of socioeconomic status in epidemiological studies.^5^ It has high reliability and validity,^5^ is generally stable after early adulthood, and shapes future occupational opportunities and income potential. A Dutch study found that persons with bipolar disorder type 1 were more likely to have completed higher education than controls,^6^ and individuals with excellent school performance were shown to have a fourfold increased risk for developing bipolar disorder compared with those with average degrees.^7^ However, a Norwegian study found a similar level of education, but lower social status, in people with bipolar disorder compared with the general population.^8^

## Socioeconomic factors and management of bipolar disorder

Besides being risk factors for disorders, socioeconomic factors might also be associated with the type of treatment patients receive.^9–12^ Inequalities of treatment because of socioeconomic status, gender or race have generally received more attention in somatic care^13,14^ than in mental healthcare, and the literature is yet sparse regarding potential treatment inequalities in relation to educational level in psychiatry, let alone bipolar disorder.

The aim of this study was to investigate if educational level is associated with treatment and management of bipolar disorder in Sweden.

## Method

### Study population

We used data from the Swedish National Quality Register for Bipolar Disorder (BipoläR), which has been described previously.^15^ BipoläR contains data on bipolar diagnosis, including subtypes, comorbid psychiatric and somatic diagnoses, outcome data (such as number of depressive, hypomanic, manic and mixed episodes during the past 12 months; compulsory institutional care; and duration of in-patient care during the past 12 months), data on treatment (drug treatment, electroconvulsive therapy (ECT) and psychological treatment) and data on psychosocial functioning. BipoläR was launched in 2004, and currently, 240 psychiatric out-patient units report to the register. The participation is voluntary for both the physician and patient. All demographic areas across Sweden, and both public and private mental healthcare providers, are represented in BipoläR. Data is typically collected by the treating physician and entered into a web-based application. After the first registration, which can occur at any time point during out-patient treatment, information is updated annually.

We extracted data from 13 304 unique individuals entered during the period 2004–2013. The reason for not including cases after 2013 is that BipoläR changed the registration form in 2014, whereupon the education variable was excluded. We included patients with bipolar disorder type 1, type 2 and not otherwise specified. We excluded individuals with schizoaffective disorder or other comorbid psychotic syndromes, to ensure that antipsychotics had been prescribed for bipolar disorder rather than for persistent psychotic symptoms. However, patients with bipolar diagnosis presenting psychotic symptoms restricted to affective episodes were included in our study population. We also excluded individuals with intellectual disabilities or autism spectrum disorders, considering that these conditions negatively affect educational level. Further, individuals with ongoing education and those younger than 22 years were excluded because they might not yet have reached their highest level of education. Finally, we excluded individuals with missing data on educational level or treatment. We analysed the remaining 10 065 individuals with bipolar disorder, of which 4289 were diagnosed with bipolar disorder type 1, 4020 were diagnosed with bipolar disorder type 2 and 1756 were diagnosed with bipolar disorder not otherwise specified.

### Exposure

The main exposure was level of education. The highest completed education level for each patient in the register was reported as follows: ‘not completed primary school’, ‘completed primary school’, ‘completed upper secondary school’ or ‘at least 2 years post-secondary education’. In Sweden, primary school is 9 years and has been mandatory since 1962. Three years of upper secondary school education is optional, but presently very common and required for many occupations. By 2014, almost half of Sweden's inhabitants aged 25–64 years had upper secondary education, more than a third had some form of post-secondary education and more than a quarter had at least 3 years of university education.^16^ In our analyses, we therefore dichotomised the education variable into ‘no university studies’ and ‘university studies’.

### Outcomes

Pharmacological treatment variables were current treatment with mood stabilisers as a group, lithium, lamotrigine, divalproex, antipsychotics as a group, quetiapine/aripiprazole/olanzapine as a group, first-generation antipsychotics (fluphenazine, flupenthixol, haloperidol, chlorpromazine, chlorprothixene, perphenazine, prochlorperazine, sulpiride, thioridazine and zuclopenthixol), antidepressants as a group, tricyclic antidepressants (amitriptyline, imipramine, clomipramine, nortriptyline, trimipramine, lofepramine and maprotiline) and benzodiazepines. Other treatment variables were having received ECT at any time, having had at least ten sessions of psychological treatment, having received psychoeducation for bipolar disorder, having had psychoeducation provided to next of kin, compulsory in-patient care and duration of in-patient care during the past 12 months. All outcome variables except duration of in-patient care were dichotomous, with the answer ‘yes’ coded as 1.

### Covariates

Demographic variables, such as age and psychosocial functioning (captured with the Global Assessment of Functioning (GAF) scale, according to the DSM-IV^17^), were included as covariates. We included additional covariates in relation to specific outcome variables. For lithium and lamotrigine, we adjusted for bipolar subtype because lithium is more likely to be used in type 1 and lamotrigine is more likely to be used in type 2. For antipsychotic treatment, we included the number of elated and mixed episodes during the past 12 months as a confounder, because manic/mixed episodes are commonly treated with antipsychotics. For antidepressant drug treatment and ECT, we included the number of depressive episodes during the past 12 months as a covariate. For benzodiazepine treatment, we added comorbid anxiety disorders as a covariate. For psychological treatment, we included comorbid personality disorders as a covariate because it is an indication for psychotherapy. All covariates except age, GAF function score and number of affective episodes were dichotomous, with the answer ‘yes’ coded as 1.

### Statistical analyses

SPSS version 22 (IBM SPSS Statistics 22.0) for Windows was used for statistical analyses. Associations between educational level and interventions were analysed with binary logistic regression. Adjusted odds ratios were computed for each outcome variable by performing two regression models. In the first regression model, we adjusted for age and GAF function score. In the second regression model, we adjusted for additional confounders as described above.

We performed two sensitivity analyses. In the first sensitivity analysis, we excluded all individuals under 26 years old, to minimise the risk for ongoing education that might change educational status. The results remained identical with our main analysis (data not shown). In the second sensitivity analysis, we stratified the study population into three groups, according to age at entering the BipoläR register (age groups 22–44, 45–64 and >64 years), because there could be an age effect with respect to educational level across generations.

### Ethics and consent statement

The study was approved by the regional Ethical Review Board in Gothenburg (approval number 294-11), and all procedures contributing to this work comply with the ethical standards of the relevant national and institutional committees on human experimentation and with the Helsinki Declaration of 1975, as revised in 2008. According to Swedish law, inclusion in Swedish quality registers follows an opt-out procedure, where patients are informed and may decline to participate, in which case data cannot be recorded. All analyses were conducted on a de-identified data-set, where neither individual patients nor physicians can be identified or traced in the material.

## Results

### Study sample and characteristics

Clinical and demographical data for the entire study sample of 10 065 patients are shown in Table 1.

**Table 1:** Clinical and demographical characteristics of 10 065 patients with bipolar disorder

	Not university	University	*P*-value
Bipolar subtype			<0.001
Type 1, % (*n*)	42.8 (2628)	42.4 (1661)	
Type 2, % (*n*)	38.6 (2373)	42.0 (1647)	
Not otherwise specified, % (*n*)	18.6 (1143)	15.6 (613)	
Female, % (*n*)	62.1 (3814)	60.9 (2389)	0.248
Age, mean (s.d.)	48.0 (15.5)	49.8 (13.9)	<0.001
Global Assessment of Functioning function score, mean (s.d.)	61.5 (19.0)	66.9 (17.5)	<0.001
Number of medications, mean	2.4 (1.4)	2.2 (1.3)	<0.001
Comorbidities, % (*n*)			
Substance misuse	5.4 (323)	4.0 (151)	0.001
Anxiety disorders	13.5 (803)	9.9 (376)	<0.001
Personality disorders	4.2 (248)	2.4 (93)	<0.001
Interventions, % (*n*)			
Mood stabilisers as a group	85.1 (5226)	85.1 (3337)	0.948
Lithium	56.2 (3451)	57.6 (2259)	0.154
Lamotrigine	25.7 (1582)	25.7 (1006)	0.918
Divalproex	10.7 (655)	9.5 (373)	0.064
Antipsychotics as a group	35.5 (2179)	31.3 (1227)	<0.001
Quetiapine/aripiprazole/olanzapine	25.1 (1540)	23.5 (923)	0.083
First-generation antipsychotics	4.8 (297)	3.4 (134)	0.001
Antidepressants as a group	45.0 (2764)	44.6 (1747)	0.671
Tricyclic antidepressants	3.2 (196)	2.3 (92)	0.008
Benzodiazepines	41.7 (1531)	39.1 (933)	0.043
Electroconvulsive therapy	21.9 (1308)	21.0 (803)	0.143
Psychotherapy	65.2 (4003)	69.0 (2705)	<0.001
Psychoeducation	21.9 (1347)	24.7 (968)	0.001
Psychoeducation to next of kin	14.6 (900)	17.3 (677)	<0.001
Compulsory in-patient care	13.9 (475)	10.3 (245)	<0.001
Duration of in-patient care, mean (s.d.), days	34.7 (40.3)	33.3 (38.3)	0.632

### Education and interventions

The educational level was not associated with treatment with mood stabilisers as a group, lithium, lamotrigine or valproic acid (Table 2).^18^ Nor were there any associations between educational level and antipsychotics as a group, or the group of quetiapine/aripiprazole/olanzapine, after adjusting for the number of elated or mixed episodes. However, the use of first-generation antipsychotics was higher in the low-education group. There was no association between the level of education and ECT or antidepressants as a group, after adjusting for the number of depressive episodes. However, tricyclic antidepressants were more common among individuals with lower education than among those with higher education. Further, there was no association between benzodiazepines and educational level after adjusting for comorbid anxiety disorders. However, higher level of education was associated with increased likelihood of having received psychoeducation, psychoeducation for next of kin and psychotherapy. Finally, individuals with lower education had a higher rate of compulsive in-patient care.

**Table 2:** Association between educational level and interventions for bipolar disorder^18^

Intervention	Model 1^a^	Model 2
	aOR^b^ (95% CI)	aOR^b^ (95% CI)
Mood stabilisers as a group	0.96 (0.86–1.07)	
Lithium	0.97 (0.89–1.05)	0.99^c^ (0.91–1.08)
Lamotrigine	1.11 (1.01–1.22)	1.08^c^ (0.98–1.19)
Divalproex	0.92 (0.81–1.06)	
Antipsychotics as a group	0.90 (0.83–0.99)	0.94^d^ (0.86–1.03)
Quetiapine/aripiprazole/olanzapine	1.01 (0.92–1.11)	1.04^d^ (0.94–1.14)
First-generation antipsychotics	0.71 (0.58–0.88)	0.76^d^ (0.62–0.94)
Antidepressants as a group	1.05 (0.96–1.14)	1.07^e^ (0.98–1.17)
Tricyclic antidepressants	0.74 (0.58–0.96)	0.76^e^ (0.59–0.97)
Benzodiazepines	0.97 (0.87–1.09)	0.99^f^ (0.88–1.10)
Electroconvulsive therapy	0.94 (0.85–1.04)	0.94^e^ (0.85–1.04)
Psychotherapy	1.31 (1.20–1.43)	1.34^g^ (1.22–1.46)
Psychoeducation	1.18 (1.07–1.30)	
Psychoeducation for next of kin	1.24 (1.11–1.38)	
Compulsory in-patient care	0.79 (0.67–0.93)	

a. Model 1 was adjusted for age and Global Assessment of Functioning function score.

b. Adjusted odds ratio (aOR) for educational level versus intervention. An aOR > 1 means that the intervention is more common in the group with higher education.

c. Model 2, adjusted for bipolar type.

d. Model 2, adjusted for number of manic, hypomanic and mixed episodes.

e. Model 2, adjusted for number of depressive episodes.

f. Model 2, adjusted for comorbid anxiety disorders.

g. Model 2, adjusted for comorbid personality disorders.

In the sensitivity analysis stratified by age, the results were in concordance with main analysis despite some results falling short of statistical significance, which is likely because of lower statistical power (see Supplementary Table 1 available at https://doi.org/10.1192/bjo.2021.19).

## Discussion

We investigated if patients’ level of education was associated with the management of bipolar disorder in a total cohort of 10 056 individuals. We found that higher education was associated with increased likelihood of receiving psychological treatment and psychoeducation, and decreased likelihood of treatment with first-generation antipsychotics and tricyclic antidepressants.^18^ Finally, compulsory in-patient care was more common in the low-education group.^18^

There are few previous studies on the importance of patients’ education for bipolar disorder management. Levine et al^19^ did not find any association between educational level and pharmacological treatment in a USA study of 457 patients with bipolar disorder type 1, who listed their medication use within the past month. Park et al^20^ found a negative correlation between higher education (college degree) and treatment with second-generation antipsychotics in bipolar disorder type 1. Not only were these studies several order of magnitudes smaller than ours, but the diverging findings could also be a result of differing healthcare organisation and inequality indices across countries. This is exemplified by a study from the USA, where higher education (college) predicted benzodiazepine use in 482 patients with bipolar disorder type 1 or 2.^21^ The authors explained this by limited coverage of benzodiazepines by some state insurance programmes. In Sweden, all citizens are covered by the same insurance programme, and we could not replicate the USA study finding. Regarding psychological interventions, three previous studies, although not limited to bipolar disorder, are in line with our findings that higher educational level is associated with higher probability of receiving psychotherapy.^22–24^

It might not be possible to fully explain the mechanisms driving inequality because research on healthcare disparities simultaneously study differences in access, need and demand, without always being able to adjust for one or the other. With this caveat, dissimilarities may arise on the system, clinician or patient level.^18^

From a systems perspective, Sweden is a welfare state, with relatively low health inequality and equal access to healthcare. Yet, inequalities in mental health and healthcare have been reported in Sweden.^25,26^ Psychiatric units in socioeconomically disadvantaged geographical areas may lack access to psychotherapists and have reduced possibility to offer the treatment, regardless of patients’ educational level.^18^

As to the role of clinicians, we are not aware of any studies suggesting that patients with higher education have a greater need for, or would respond better to, psychological treatments. Conversely, we are not aware of any studies suggesting that patients with lower education would respond better to first-generation antipsychotics or tricyclic antidepressants. Interestingly, it has been suggested that the patient and doctor being on the same socioeconomic level could influence a doctor's choice for drug prescription.^27^

With respect to the role of patients, expectations and ability to demand a specific treatment have been shown to be related to the patient's educational level, and might drive differences in psychological treatments.^9^ Our finding that higher-educated patients with bipolar disorder received fewer first-generation antipsychotics and tricyclic antidepressants could also be ‘patient-mediated’, as persons with higher education may have better access to drug information, including side-effects.^18^ Our finding of an inverse relationship between use of first-generation antipsychotics and educational level has also been demonstrated in a recent study in elderly patients with and without dementia in Sweden.^11^ Finally, educational level might be associated with the severity or type of illness, which in turn, warrants different interventions. For example, higher educational level is a proxy for cognitive reserve, which has been suggested to be associated with course and functional outcome of bipolar disorder.^28^ Although our regression models were adjusted for GAF function score, number of mood episodes and comorbid personality disorder, we could not control for differences in cognitive function or the severity of the disorder.^18^

It is worth considering that the drug treatment disparities we found might be attributable to differences in the income rather than educational level *per se*, as educational level reflects socioeconomic position. Swedish public services do not meet the need and demand for psychological treatment, and patients with higher education might have the financial means to afford private psychotherapy.^18^ Unfortunately, we cannot tell if psychotherapy was provided by private or public providers. Psychoeducation is, however, provided by publicly funded psychiatric units in Sweden, and the difference we found is unlikely to be explained by income differences. With respect to drug treatment, new drugs tend to be more expensive, and have been shown to be prescribed more frequently in higher socioeconomic groups.^10^ However, income differences across educational groups are less likely to explain our findings, not only because socioeconomic differences in Sweden are modest from an international perspective, but also because drug treatment is highly subsidised: after an initial cost of 2300 Swedish kronor (approximately US$240 as of February 2019), patients are eligible for free prescribed drugs for the remainder of a 12-month period, through the social welfare system.^18^ This suggests that the disparities in prescription drugs we find are likely to be associated with educational level rather than income.^18^ In support of this notion, Nordin et al^12^ found a positive relationship between education and drug utilisation, after controlling for income.

### Strengths and limitations

We studied a large, representative real-world clinical cohort of Swedish patients with bipolar disorder. Among limitations that need to be considered is the cross-sectional study design, which does not allow causal inference. We further lacked information on the indication for drug prescriptions (e.g. mood stabilisers, antipsychotic medication). To partially address this, we excluded individuals with psychotic conditions, including schizoaffective disorder, and we also adjusted for bipolar subtype, comorbid psychiatric conditions and number of depressive or elated episodes. Third, although registry-based bipolar diagnoses in Sweden have been shown to have good validity overall,^29^ and the BipoläR diagnoses are made according to the DSM-IV, diagnoses were made in clinical routine and not according to a research protocol. On the other hand, this procedure reflects real-world praxis. Finally, BipoläR do not include all patients with bipolar diagnosis in Sweden; however, BipoläR has previously been found to be representative to the whole Swedish bipolar disorder population,^30^ and the gender distribution and education levels in our study sample are similar to the general bipolar disorder population in Sweden and to other large bipolar disorder study samples.^16,31^

In conclusion, we find that the level of education is associated with differences in bipolar disorder management in Sweden.^18^ We fail to identify any medical rationale behind these differences, which thus may indicate unequal treatment.^18^ To disentangle the mechanism underlying these disparities, studies would need to include data on specific indications for pharmacological treatments, geographical differences in access to care, and patients’ and clinicians’ attitudes.^18^

## Data Availability

The data-sets generated and/or analysed during the current study are not publicly available because of the Swedish law for register data, but are available from the corresponding author, A.K., on reasonable request.

## Supplementary material

For supplementary material accompanying this paper visit http://dx.doi.org/10.1192/bjo.2021.19. click here to view supplementary material
