# Supplementary material for: Patient educational level and management of bipolar disorder
Source: BJPsych Open. 2021 Mar 8;7(2):e63. doi: 10.1192/bjo.2021.19 (PMC8058931; doi:10.1192/bjo.2021.19)
Supplement: Supplementary file 1 [file S2056472421000193sup001.docx]

**Supplement**

**Table 1. Association between educational level and treatment for patients stratified by age**

| **Intervention** |  | **aOR^1^ (95% CI)** |  |
| --- | --- | --- | --- |
|  | **22-44 years of age** | **45-64 years of age** | **Older than 64 years of age** |
| Mood stabilizers as a group | 0.92 (0.77-1.10) | 0.96 (0.81-1.15) | 1.11 (0.80-1.52) |
| Lithium ^a)^ | 0.94 (0.82-1.08) | 1.05 (0.92-1.20) | 1.00 (0.80-1.25) |
| Lamotrigine ^a)^ | 0.99 (0.86-1.14) | 1.07 (0.92-1.24) | 1.25 (0.92-1.68) |
| Divalproex ^b)^ | 0.87 (0.70-1.08) | 0.92 (0.76-1.12) | 0.87 (0.60-1.25) |
| Antipsychotics as a group ^c)^ | 1.01 (0.88-1.17) | 0.85 (0.74-0.97) | 0.80 (0.64-1.02) |
| Quetiapine/aripiprazole/olanzapine ^c)^ | 1.04 (0.90-1.21) | 1.02 (0.88-1.18) | 0.89 (0.68-1.16) |
| First-generation antipsychotics ^c)^ | 0.73 (0.48-1.12) | 0.63 (0.47-0.85) | 1.11(0.68-1.80) |
| Antidepressants as a group ^d)^ | 0.99 (0.87-1.14) | 1.07 (0.94-1.22) | 1.13 (0.91-1.39) |
| Tricyclic antidepressants ^d)^ | 0.62 (0.37-1.05) | 0.81 (0.57-1.14) | 1.13 (0.91-1.39) |
| Benzodiazepines ^e)^ | 0.99 (0.82-1.19) | 0.98 (0.83-1.15) | 0.92 (0.70-1.20) |
| ECT ^d)^ | 1.04 (0.85-1.26) | 0.97 (0.84-1.13) | 0.73 (0.59-0.91) |
| Psychotherapy ^f)^ | 1.08 (0.93-1.26) | 1.46 (1.27-1.68) | 1.23 (1.00-1.52 |
| Psychoeducation ^b)^ | 1.24 (1.07-1.43) | 1.06 (0.92-1.23) | 1.04 (0.78-1.40) |
| Psychoeducation for next-of-kin ^b)^ | 1.28 (1.08-1.50) | 1.18 (0.99-1.40) | 1.04 (0.74-1.48) |
| Compulsory inpatient care ^b)^ | 0.75 (0.58-0.96) | 0.80 (0.62-1.03) | 0.89 (0.55-1.45) |

^1^ aOR = Adjusted odds ratio for educational level vs intervention. An aOR>1 means that the intervention is more common in the group with higher education.

^a)^ adjusted for age, GAF-function, and bipolar type; ^b)^ age, and GAF-function; ^c)^ age, GAF-function, and number of manic, hypomanic and mixed episodes; ^d)^ age, GAF-function, and number of depressive episodes; ^e)^ age, GAF-function, and comorbid anxiety disorders; ^f)^ age, GAF-function, and comorbid personality disorders.
